# Use of GLP-1 Receptor Agonists is Associated with an Increased Rate of Upper Eyelid Blepharoplasty

**DOI:** 10.21203/rs.3.rs-9665910/v1

**Published:** 2026-05-26

**Authors:** Victor F. A. Almeida, John Ha, Fernando Duraes, Manoela Dantas, Ahmed M. Hashem, Risal S. Djohan, Eliana F. R. Duraes

**Affiliations:** Cleveland Clinic; Cleveland Clinic; Cleveland Clinic; Cleveland Clinic; Cleveland Clinic; Cleveland Clinic; Cleveland Clinic

**Keywords:** Blepharoplasty, GLP-1 receptor agonist, Ozempic face, Tirzepatide, Semaglutide

## Abstract

**Background:**

Glucagon-like peptide-1 receptor agonists (GLP-1 RA) have transformed the management of obesity and type 2 diabetes. Emerging reports describe accelerated facial soft-tissue deflation and periorbital changes among treated patients, often referred to as “Ozempic face.” Given the thin skin and minimal subcutaneous tissue of the upper eyelid, rapid weight loss may exacerbate dermatochalasis and increase demand for surgical correction. This study evaluates whether GLP-1 RA therapy is associated with an increased rate of upper eyelid blepharoplasty.

**Methods:**

A retrospective cohort study was conducted using the TriNetX Research Network. Female adults aged 18 or older with diabetes mellitus and essential hypertension between January 2015 and January 2020 were identified. Patients prescribed GLP-1 RA comprised the exposure cohort; patients without GLP-1 RA served as controls. Follow-up extended up to five years. The primary outcome was upper eyelid blepharoplasty. Propensity score matching (1:1 nearest neighbor) was performed for age, race, BMI, and comorbidities. Kaplan–Meier survival analysis and Cox proportional hazards modeling were performed. A secondary analysis compared patients achieving weight loss without GLP-1 RA to matched controls with stable BMI.

**Results:**

A total of 821,792 eligible female patients were identified, of whom 28,970 received GLP-1 RA therapy. After propensity score matching, 23,349 patients remained in each cohort. At five-year follow-up, 96 GLP-1 RA users (0.41%) underwent blepharoplasty compared with 36 controls (0.15%). GLP-1 RA use was associated with an increased hazard of blepharoplasty (hazard ratio 2.22; 95% confidence interval 1.51–3.26; p < 0.001). Kaplan–Meier analysis demonstrated shorter time to surgery among GLP-1 RA users. In the secondary analysis, non-GLP-1 RA weight loss was not associated with increased blepharoplasty incidence (hazard ratio 1.31; 95% confidence interval 0.89–1.93; p = 0.56).

**Conclusions:**

GLP-1 receptor agonist use is associated with a significantly increased rate and earlier timing of upper eyelid blepharoplasty compared with matched controls. Weight loss alone did not demonstrate the same association, suggesting a distinct pattern of facial remodeling related to GLP-1 RA therapy. As pharmacologic weight loss expands, plastic surgeons should anticipate increased demand for periorbital rejuvenation and counsel patients accordingly.

## Introduction

Glucagon-like peptide-1 (GLP-1) receptor agonists (RA) have transformed the management of obesity and type 2 diabetes, providing a pharmacologic alternative to bariatric surgery for achieving substantial and sustained weight loss [18]. Since the approval of agents such as semaglutide and liraglutide, global prescription rates have risen exponentially, with over 9 million prescriptions written in the final three months of 2022 alone [19]. These medications mimic the action of the endogenous incretin GLP-1 by enhancing insulin secretion, delaying gastric emptying, and promoting satiety, resulting in an average weight reduction of 10–15% of baseline body weight within the first year of therapy [20, 21]. While these effects represent a major advance in metabolic care, they have also introduced new aesthetic and reconstructive considerations, particularly due to the rapid and visible changes in body composition [1,7,10].

The face is especially sensitive to variations in soft-tissue volume and skin elasticity. Rapid depletion of subcutaneous fat may accentuate age-related features such as midfacial deflation, periorbital hollowing, and upper eyelid laxity, an effect widely popularized in the media as “Ozempic face” [2, 5, 6]. In parallel, aesthetic and dermatologic practices have reported increasing demand for facial rejuvenation procedures among patients using GLP-1 receptor agonists [1, 4]. A recent multicenter study has shown a sharp rise in aesthetic surgery among these patients, exceeding the growth seen in post-bariatric populations. [4]. Body-contouring procedures such as panniculectomy, brachioplasty, and breast surgery have been most affected, suggesting that pharmacologic weight loss has become a new driver of aesthetic intervention [4,11].

However, the influence of GLP-1 receptor agonists on facial surgical procedures remains poorly explored, and data specifically examining blepharoplasty are particularly scarce [1,7]. Understanding this relationship is important because the upper eyelid has the thinnest skin on the face and minimal subcutaneous tissue, making it highly susceptible to laxity, dermatochalasis, and contour loss [9,14–16]. As a result, the periorbital region is among the most volume-sensitive areas of the face and one of the first to exhibit visible changes associated with weight loss and aging, leading to both aesthetic and functional impairments such as eyelid heaviness and visual field obstruction [6,14–16]. Moreover, evidence suggests that GLP-1 RA may induce a distinct pattern of facial aging characterized by preferential depletion of superficial fat and reduced regenerative activity of adipose tissue [5, 6, 29–31]. In this context, identifying potential relationships between GLP-1 receptor agonist use and blepharoplasty incidence may help surgeons better understand how these medications influence facial soft-tissue dynamics and aging patterns, while also enabling them to anticipate patient expectations and optimize surgical planning in this growing population.

Therefore, this study aims to evaluate the association between GLP-1 receptor agonist use and the incidence of upper eyelid blepharoplasty in a large, multi-institutional cohort. We hypothesized that patients treated with GLP-1 receptor agonists would demonstrate a higher rate and shorter time to blepharoplasty compared with matched controls with similar comorbidities.

## Methods

### Study Design and Data Source

We conducted a retrospective cohort study using the TriNetX Research Network in accordance with the Strengthening the Reporting of Observational Studies in Epidemiology (STROBE) guidelines. TriNetX aggregates deidentified electronic health records from more than 130 million individuals across 111 healthcare organizations (HCOs) within the Research network. The analytic workspace was created on October 7, 2025. Grammar and phrasing assistance during manuscript preparation was provided by Claude (Anthropic).

### Study Population

Eligible participants were female adults aged 18 years or older diagnosed with both diabetes mellitus (ICD-10-CM E08-E13) and essential hypertension (I10). Cohort A included patients who received GLP-1 receptor agonists, identified using Anatomical Therapeutic Chemical classification system (ATC class A10BJ), between January 1, 2015, and January 1, 2020. The GLP-1 receptor agonist drugs available prior to 7/1/2020 included exenatide, liraglutide, dulaglutide, lixisenatide, and semaglutide. Cohort B included similarly eligible patients who did not receive GLP-1 RA therapy during the same period. Patients were excluded if their index events occurred more than 20 years before analysis initiation.

### Index Event and Follow-Up

For Cohort A, the index event was defined as the first recorded prescription or administration of a GLP-1 RA between January 1, 2015, and January 1, 2020. For Cohort B, the index event was the first ambulatory visit recorded in that same period. Follow-up began one day after the index event and continued until the earliest of five years post-index or the patient's last recorded encounter in TriNetX, at which point observation was censored.

### Outcomes

The primary outcomes were cosmetic surgical procedures performed during follow-up. We used Current Procedural Terminology (CPT) codes and assessed these through TriNetX standardized terminology mapping. Our outcomes included upper eyelid blepharoplasty (CPT 15823).

### Statistical Analysis

We used 1:1 greedy nearest-neighbor propensity score matching (PSM) without replacement to balance cohorts across demographic, clinical, and laboratory characteristics. Variables included age at index, race, body mass index (BMI), diabetes mellitus, essential hypertension, chronic kidney disease, hyperlipidemia, chronic obstructive pulmonary disease, and medication use (metformin, insulin, sodiumglucose cotransporter 2 [SGLT2] inhibitors). Balance was assessed by standardized mean differences (SMD), with SMD < 0.10 considered acceptable.

Following matching, we conducted risk analyses to estimate absolute risks, risk ratios, and odds ratios for each outcome. Kaplan-Meier survival curves were used to estimate time-to-procedure, and log-rank tests compared survival distributions between cohorts. Cox proportional-hazards models provided hazard ratios (HR) and 95% confidence intervals (CI). Continuous variables were compared with twosample t-tests, and categorical variables with chi-squared tests. Statistical significance was set at twotailed p < 0.05. All analyses were conducted within the TriNetX analytical environment, which automatically censors follow-up at the last recorded encounter or at five years post-index.

## Results

### Demographics

A total of 821,792 female patients aged 18 years or older with diagnoses of diabetes mellitus and essential hypertension were identified across participating healthcare organizations. Among these, 28,970 had received a GLP-1 RA prescription between January 1, 2015, and January 1, 2020. After 1:1 PSM, 23,349 patients remained in each cohort (GLP-1 RA vs. non–GLP-1 RA).

Before matching, significant imbalances were observed between groups in age, comorbidities, and medication use (all p < 0.001). Following matching, all standardized mean differences were less than 0.10, indicating acceptable covariate balance across demographic, clinical, and laboratory variables (Supplemental Table 1). Baseline BMI was slightly lower among GLP-1 RA users (30.7 ± 5.2) compared with controls (31.2 ± 6.5) after matching.

### Outcomes

**Figure 1:**
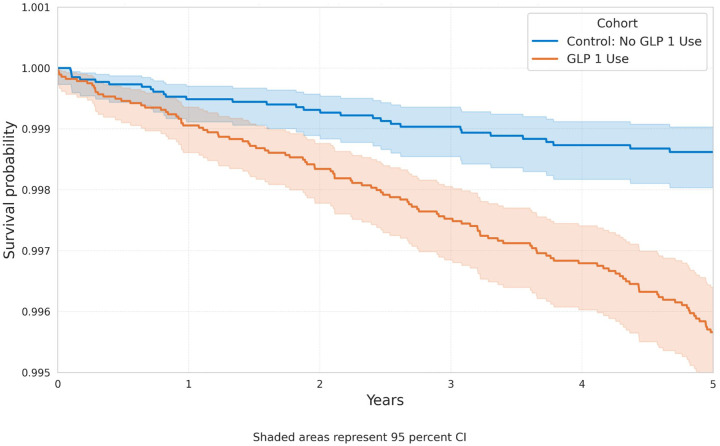
Kaplan–Meier survival curve comparing time to upper eyelid blepharoplasty between GLP-1 receptor agonist users and controls. - **Note**: GLP-1 receptor agonist users exhibited a shorter time to blepharoplasty compared with matched controls (log-rank p < 0.001).

At five-year follow-up, mean BMI declined further among GLP-1 RA users (27.97 ± 4.18) relative to controls (29.90 ± 5.89). GLP-1 RA users exhibited a significantly higher incidence of both upper eyelid blepharoplasty compared with matched controls. The hazard ratio for upper eyelid blepharoplasty was 2.22 (95% CI, 1.51–3.26; p < 0.001) (Table 1, Fig. 1).

### Secondary Analysis

A secondary analysis compared patients who achieved weight loss without GLP-1 RA therapy to matched controls with stable BMI. After 1:1 PSM, 24,690 patients remained in each group. Baseline BMI (Supplemental Table 2) was similar between groups (34.1 ± 4.9 vs. 33.8 ± 3.3) and remained lower in the weight-loss group at follow-up (31.68 ± 5.16 vs. 33.62 ± 2.22, Table 2). In the weight-loss analysis, there was no significant difference in the incidence or timing of upper eyelid blepharoplasty (HR 1.31; 95% CI, 0.89–1.93; p = 0.56) (Table 2).

## Discussion

In this large retrospective cohort study of more than one million female patients with diabetes and hypertension, we found that the use of GLP-1 receptor agonists was associated with a significantly higher rate of upper eyelid blepharoplasty compared with matched nonusers. After 1:1 PSM, 23,349 patients remained in each cohort, ensuring adequate balance across demographic and clinical characteristics. The incidence of blepharoplasty was 0.41% among GLP-1 RA users versus 0.15% in controls, corresponding to a hazard ratio of 2.22 (95% CI, 1.51–3.26; p < 0.0001). These results suggest that these patients underwent blepharoplasty both earlier and more frequently after initiating therapy.

These findings are consistent with recent evidence demonstrating a broader rise in aesthetic procedures among individuals using GLP-1 receptor agonists [4, 5, 10]. In a multicenter TriNetX analysis, the annual growth rate of aesthetic surgery among GLP-1 RA users exceeded 50% after 2021, surpassing that observed in post-bariatric surgery patients [4]. Another study found significant correlations between GLP-1 RA use and body contouring procedures, including brachioplasty (r = 0.23), panniculectomy (r = 0.21), and breast surgery (r = 0.28), with overall surgery rates markedly higher among pharmacologic weight-loss users [11].

GLP-1 receptor agonists mimic the action of the incretin hormone glucagon-like peptide-1, enhancing insulin secretion, suppressing glucagon release, and delaying gastric emptying [19]. These mechanisms promote satiety, reduce caloric intake, and result in significant and sustained weight loss, typically 10–15% of baseline body weight within the first year of therapy [20, 21,28]. While such results have transformed the management of obesity and metabolic disease, the rapidity of weight reduction can generate aesthetic consequences [1,5]. The face is particularly sensitive to small changes in soft-tissue volume, and rapid loss of subcutaneous fat may accentuate signs of aging such as midfacial deflation, periorbital hollowing, and skin laxity [2,5,6,9,14–16]. These visible effects, often labeled in popular media as “Ozempic face,” have increased public awareness and may drive more patients to seek corrective aesthetic procedures, including blepharoplasty [10].

From an anatomical standpoint, it is expected that patients who experience rapid weight loss would be more likely to seek eyelid surgery. The upper eyelid has the thinnest skin on the face and minimal subcutaneous tissue, making it highly susceptible to laxity and dermatochalasis when facial fat and skin elasticity decline. Rapid fat depletion and changes in dermal quality associated with GLP-1 RA therapy can accentuate these structural vulnerabilities, leading to redundant eyelid skin, pseudoherniation of orbital fat, and descent of the brow and midface—all of which contribute to a fatigued or aged appearance [1,5,6,7, 14, 15, 16]. Blepharoplasty addresses these issues by removing excess skin and repositioning or excising orbital fat to restore a more youthful contour. Therefore, patients treated with GLP-1 receptor agonists may present these aesthetic and functional concerns earlier or more prominently than the general population, contributing to a higher demand for upper eyelid blepharoplasty. [1,6,7].

A recent imaging-based study using three-dimensional facial analysis confirmed that patients treated with GLP-1 receptor agonists exhibit measurable loss of soft-tissue volume in the midface and periorbital regions, with predominant reduction in superficial fat compartments rather than deep ones [6]. Specifically, patients experienced a median 9% reduction in total midface volume (11% in superficial fat and 7% in deep fat), and regression analysis indicated a 7% loss of facial volume for every 10 kg of weight loss [6]. This quantitative evidence underscores the unique pattern of fat redistribution seen with GLP-1 RA therapy—superficial (subcutaneous) facial fat pads are preferentially depleted relative to deeper compartments, accompanied by loss of dermal and subcutaneous white adipose tissue and diminished facial muscle mass [5, 6, 22]. In vitro studies have shown that GLP-1 agents can directly affect adipose-derived stem cells, reducing their proliferation and ability to differentiate into mature adipocytes [5, 29, 30, 31]. This alteration weakens the regenerative function of facial fat and decreases the release of growth factors and cytokines from dermal white adipose tissue, disrupting normal paracrine signaling with fibroblasts. As a result, collagen synthesis and dermal repair are impaired, contributing to skin thinning, reduced elasticity, and accelerated facial aging [5, 29, 30, 31]. This contrasts with normal aging, which primarily involves deep-fat resorption and bony remodeling with relative preservation of superficial fat until later decades [23, 24], producing a distinct aesthetic and metabolic profile. Moreover, systematic reviews of massive weight-loss patients report pronounced deficits along the mid-cheek and central neck, with deeper nasolabial folds and malar flattening that can make individuals appear several years older [9]. These regional patterns should be recognized when counseling GLP-1 RA users about expected changes and surgical planning [9].

The biological mechanisms linking GLP-1 receptor agonists to these changes remain incompletely understood. Most evidence suggests an indirect effect through overall caloric deficit and accelerated lipolysis rather than direct adipocyte modulation [5,7]. However, rapid GLP-1-driven weight loss may also involve loss of lean body mass—a systematic review indicates that approximately 39–40% of the weight lost is fat-free mass, contributing to diminished facial musculature [5,21,28]. Moreover, hormonal changes also accompany rapid weight reduction: declines in estrogen and dehydroepiandrosterone reduce collagen and elastin synthesis, compromising skin elasticity [5]. Thus, facial deflation reflects not only fat loss but also muscle atrophy and altered extracellular-matrix metabolism [5]. From a psychosocial standpoint, patients may perceive that they look up to five years older and experience distress over hollowed cheeks, temples and jawlines [9]. By contrast, bariatric surgery induces a more extensive and rapid reduction in body weight; for example, a recent U.S. cohort study reported a mean weight loss of 28.3% at two years following metabolic bariatric surgery (MBS), compared with 10.3% for GLP-1 receptor agonists (GLP-1 RAs) [25]. In distinction from GLP-1–related weight-loss remodeling, existing evidence shows that metabolic bariatric surgery produces substantial reductions in both superficial and deep fat compartments throughout the body, including the face, where clinical and histological studies consistently demonstrate global deflation, skin laxity, and remodeling of facial fat layers [26–27]. Yet, despite the greater weight loss typically achieved by bariatric patients, the expanding use of GLP-1 receptor agonists encompasses a much broader and more diverse population, making such cases increasingly common in plastic surgery and therefore particularly relevant to practicing surgeons.

From a surgical perspective, understanding these mechanisms is crucial. Patients treated with GLP-1 receptor agonists may present with thinner subcutaneous tissue, decreased midfacial projection, and mild asymmetries [5,6,7,8]. Surgeons should be cautious when planning blepharoplasty or other rejuvenation procedures in this population, as further weight loss may alter postoperative outcomes. Optimal timing for surgery should be after weight stabilization, and adjunctive measures such as autologous fat grafting, dermal fillers, or energy-based skin tightening may enhance results [1,5,7,9,10]. Evidence indicates that fillers alone may create an “overfilled” appearance when correcting large volume deficits [12]. In such cases, autologous fat transfer or composite fat grafting can provide both volumizing and regenerative benefits [7]. Non-surgical approaches such as radiofrequency microneedling, high-intensity focused ultrasound, and fractional CO_2_ laser treatments can also be beneficial by promoting collagen and elastin synthesis [7, 9]. For more advanced tissue laxity, surgical interventions may range from limited blepharoplasty to extended superficial musculoaponeurotic system (SMAS) facelifts or deep-plane facelifts [9]. Comprehensive preoperative counseling should address expectations, possible recurrence of skin laxity, and the potential for continued changes with long-term GLP-1 RA use [1,7,8,9]. Furthermore, perioperative management should include discontinuing daily GLP-1 RA formulations one day before surgery and weekly formulations one week before to mitigate aspiration risk [9], along with ensuring nutritional optimization and weight stability [7].

Our study has limitations. As a retrospective analysis of a large clinical database, causality cannot be inferred. Medication type, dosage, and duration of therapy were unavailable, and weight change magnitude was not documented, preventing direct comparison of weight variation between groups and highlighting the need for further research. The reliance on procedural codes may lead to under- or overestimation of true blepharoplasty rates, and photographic or imaging data were not available to confirm objective facial changes. Additionally, our findings pertain primarily to female patients with metabolic comorbidities and may not generalize to other populations. It is also worth noting that GLP-1 receptor agonists are costly, and utilization may be concentrated among individuals with greater financial means or insurance coverage [13]. These socioeconomic disparities could confound our findings, as patients who can afford GLP-1 RA therapy may also have greater access to elective plastic surgery.

Future prospective studies incorporating imaging, biochemical, and histologic evaluation will be essential to clarify the mechanisms underlying these observations. Additionally, comparative research examining regional fat-loss patterns with caloric restriction, exercise, bariatric surgery and GLP-1 RA pharmacotherapy could clarify how various interventions reshape facial and body fat distribution, thereby better guiding aesthetic surgical planning.

## Conclusion

In conclusion, our study demonstrates a higher prevalence of upper eyelid blepharoplasty among patients using GLP-1 receptor agonists compared with matched controls. This finding is consistent with a growing body of evidence linking pharmacologic weight loss to increased aesthetic surgery demand. The likely contributors include midfacial and periorbital fat loss, reduced dermal elasticity, and heightened self-awareness influenced by public discourse surrounding “Ozempic face.” As GLP-1 RA continue to reshape the landscape of weight management, plastic surgeons should anticipate increased demand for rejuvenation procedures, counsel patients about timing and tissue quality, and remain aware of the unique anatomical and metabolic considerations associated with these agents.

## Supplementary Material

Supplementary Files

This is a list of supplementary files associated with this preprint. Click to download.

- SupplementalTable1.docx

## Figures and Tables

**Table 1:** Surgical procedures at five-year follow-up compared between GLP-1 RA users and matched controls

Procedure	Control, n (%)	GLP-1 RA, n (%)	Hazard Ratio	95% CI	p value
Upper eyelid blepharoplasty	36 (0.15)	96 (0.41)	2.22	1.51–3.26	<0.0001
Index date BMI	31.2 ± 6.5	30.7 ± 5.2	–	–	–
BMI	29.9 ± 5.9	28.0 ± 4.2	–	–	–

• **Note**: GLP-1 RA users demonstrated a significantly higher incidence of upper eyelid blepharoplasty compared with matched controls at five years post-index. BMI is presented as mean ± SD.

**Table 2:** Surgical procedures at five-year follow-up compared between weight-loss group and non-weight loss control group.

Procedure	Control, n (%)	Weight loss, n (%)	Hazard Ratio	95% CI	p value
Upper eyelid blepharoplasty	55 (0.22)	49 (0.20)	1.31	0.89 – 1.93	0.5559
Index date BMI	33.8 ± 3.3	34.1 ± 4.9	–	–	–
BMI	33.6 ± 2.2	31.7 ± 5.6	–	–	–

• **Note**: Weight loss patients demonstrated a similar incidence of upper eyelid blepharoplasty compared with matched controls with no weight loss at five years post-index. BMI is presented as mean ± SD.

## Data Availability

The datasets generated and/or analysed during the current study are not publicly available because access to the TriNetX Research Network requires an institutional license agreement, but are available from the corresponding author on reasonable request.
